# Kansas Veterinary Medical Association

**Published:** 1890-12

**Authors:** L. R. Brady

**Affiliations:** Sec’y.


*Kansas Veterinary Medical Assvciation.*—The Kansas Veterinary Medical Association met at the Copeland, in Topeka, September 18th, 1890. Meeting called to order by President Geo. C. Pritchard. Minutes of last meeting were read and approved. Election of officers resulted in the election of Geo. C. Pritchard, of Topeka, Kansas, Pres.; S. L. Hunter, of Ft. Leavenworth, Vice Pres.; L. R. Brady, or Manhattan, Sec'y, and W. H. Richards, of Emporia, Treas.

The Board of Censors elected were F. H. Cook, of Hutchinson; John Ernst, of Topeka, and S. C. Orr, of Manhattan. New members elected were Drs. Glass, Cook, Sihler and Ernst. Two papers were read, one by D. C. Ayer, of Leavenworth, a very well prepared article on "Fistula," which was well discussed, the other being an essay on "Azoturia," by Dr. Orr, of Manhattan. This was a very well prepared essay and created quite a lively discussion.

Papers to be read at the next meeting are by Drs. Daniel Lemay, of Ft. Riley, S. E. Phillips, of Wichita, and F. W. Cook, of Hutchinson. Meeting adjourned to semi-annual meeting at Wichita, second Tuesday in March.

L. R. Brady, *Sec'y.*

